# Training path of big data management and application talents based on BERTopic-TOPSIS model

**DOI:** 10.1371/journal.pone.0334127

**Published:** 2025-12-01

**Authors:** Yan Li, Tao Huang, Xiang Li

**Affiliations:** School of Management, Xi’an Polytechnic University, Xi’an, China; Alexandria University Faculty of Nursing, EGYPT

## Abstract

This study examines the discrepancy between big data talent training and industry demand. The study analyzed 85 training programs and over 10,000 job postings from two job boards in China (51job and Zhaopin). Using content analysis, social network analysis, and the BERTopic-TOPSIS model, it mined implicit information from training programs and labeled key competencies in job descriptions. A key finding was a significant supply-demand misalignment: while “data application ability” was a stated goal in 52% of programs, only 11% of graduation requirements specified concrete, measurable skills to achieve it. The study identified three primary employment pathways for big data management and application majors: data management, data analysis, and data platform development. Institutions such as Peking University and Hefei University of Technology were identified as best practices. The study then delineated a cultivation path for the major by integrating the characteristics of these employment pathways, and optimised general knowledge and compulsory courses, core courses, graduation requirements, and the cultivation objectives of the major.

## 1. Introduction

With the advent of technologies such as big data and artificial intelligence, the global volume of data has increased exponentially, ushering in the era of big data and bringing about significant changes in various industries. Since the term ‘big data’ first appeared in a Chinese government report in 2014,the emergence of data as a new factor of production in the digital age has made big data development a strategic imperative for China. To ensure the effective implementation of the big data strategy, the Ministry of Industry and Information Technology (MIIT) and the China Academy of Information and Communications Technology (CAICT) have successively released the “14th Five-Year Plan” for the development of the big data industry, the “White Paper on Big Data (2022)” and other documents, which comprehensively deployed the landing of the big data policy, put forward the problem of the lack of integrated big data elites in China, and emphasised that China urgently needs to strengthen the training of big data talents to accelerate the cultivation of big data talents. In this context, data-driven application management oriented big data talents effectively drive the rapid economic, social and industrial development, and the corresponding big data management and application specialisation has become a hot speciality applied by domestic universities.

According to the Ministry of Education’s announcement of the results of applications and approvals for undergraduate programmes at ordinary higher education institutions, a total of 184 universities have opened across the country as of 2022. The construction of big data management and application specialisation has gradually become a research hotspot [1]. However, universities are facing the common problems of professional construction, such as the irrational setting of training programmes [2], the phenomenon of “two skins” between talent cultivation and talent demand [3], and the lack of a reasonable feedback mechanism for cultivation [4]. This has led many to discuss and debate the following issues. The above issues have triggered extensive discussions among many scholars, and at present, the research on talent cultivation for big data professionals mainly focuses on three aspects.

One is to study the current status of talent cultivation from the starting point of the existing talent cultivation of big data professionals, to identify cultivation problems and to propose measures to solve them (talent cultivation side study). For example, scholars such as Zhao et al. [5] uses content analysis to mine and analyze the cultivation objectives and curriculum system of big data management and application majors, derive the cultivation status and cultivation problems of the majors, and provide talent cultivation suggestions to universities; Chen and Wei [6] explores the characteristics of the Right River Medical College of Nationalities in relation to the professional declaration, professional characteristics, and professional faculty, as well as in other aspects of exploration. The objective of this exploration is to establish professional training objectives, training processes, and curricula, as well as other personnel training system content. Sun and Yu [7] constitutes an exhaustive analysis of the prevailing issues and characteristics of the professional talent training industry. Employing a systematic approach, the study examines the development trends in talent demand and the financial management and application of big data in the context of professional talent training by financial colleges and universities. Jia [8] has selected big data management and application as the object of study, and has analysed the status quo of talent cultivation in this field. He has also proposed an innovation strategy for talent cultivation in big data management and application, to be implemented in the context of the ‘Internet+’ initiative. Yang [9] proposes a novel approach to talent training, underpinned by the OBE-CDIO concept. This approach aims to furnish a robust theoretical foundation for cultivating high-calibre data management and application talents. Yang [10] proposed a novel educational model for the joint training of industry and academia in the domain of big data management and application. This model was developed within the broader context of the new liberal arts. Liu [11] applies OBE teaching concept and CDIO education model to innovate and reform the talent cultivation mode of big data management and application, and explores the talent cultivation mode which is suitable for professional characteristics and adapts to the needs of the times. The objective of this exploration is to establish professional training objectives, training processes, and curricula, as well as other personnel training system content. Notably, the challenge of cultivating talent that meets industry demands is a global concern. For instance, international studies have also employed innovative methods to analyze and inform educational models. Park [12] emphasizes a PBL hands-on oriented healthcare data science curriculum as an educational model, the curriculum and results can serve as the cornerstone data for efficient big data analytics and data science education applicable to other universities.

The second is to mine recruitment information to derive talent demand tendency, so as to provide feedback suggestions to universities (talent demand side research). This aspect of research is mainly proposed by scholars: the talent training model lacks an effective response to the demand of the talent market [13], and needs a kind of big data talent demand research from the employer’s perspective [14]. Therefore, some scholars turn the research perspective to the talent demand side, such as Zhou and Yin [15] uses network survey method to conduct research on the type of big data management talent demand, knowledge background, comprehensive quality and ability, effectively analyse the knowledge and ability structure of big data management talent, and finally put forward the talent cultivation suggestions to universities; This approach of deriving talent requirements from job market data is also reflected in international research. De Mauro et al. [16] takes the network recruitment data as the object, and use the LDA model to identify the big data management talent demand. Using the LDA model to identify the knowledge domains and skills required by big data professionals, and in-depth analysis of the knowledge skills required by this type of talent through ability mapping. Thirdly, the status quo of big data talent cultivation and the focus of talent demand have been studied at the same time, and a link has been constructed between the two, and the difference between talent supply and demand has been finally found and talent cultivation feedback suggestions have been put forward to universities based on the difference (cultivation feedback study). The representative ones are: Wang et al. [1] analyzed the curriculum system and content of practical teaching links of big data majors of famous universities at home and abroad, and conducted network surveys on the recruitment information of big data talents of famous enterprises at home and abroad, and based on the comparative analysis of the former two, finally came up with the key points and difficulties in the process of building big data management and application majors, and put forward the improvement methods. Li et al. [17] analyses the main areas of big data capability training and the needs of the Chinese market for various kinds of data science talents. It then discusses the curriculum design process of the Data Science & Big Data Technology bachelor’s degree programme, and summarises some detailed approaches to improve the teaching experiment.

In summary, current scholars have carried out research on the issue of big data professional construction from different perspectives and to different degrees, and formed a quantitative research paradigm combining the analysis of the text of the professional training programme and the mining of social recruitment information. However, due to the late introduction of the major of big data management and application has resulted in the research on the training mode of the major being in the preliminary exploration stage. There is a paucity of quantitative research that takes into account the supply of and demand for talents at the same time. This lack of research limits the accuracy and effectiveness of universities in training big data professionals. It also has the effect of causing blindness and challenges in talent recruitment and job matching in the industry. Therefore, it has become an urgent need for the current research and practice in this field to deeply explore the talent cultivation mode of big data management and application majors, and to build a quantitative research framework that can comprehensively reflect the needs of both talent supply and demand.

The initial section of this study provides a comprehensive introduction to the research structure and methodology. The subsequent section explores the commonalities of the talent cultivation model. The third section undertakes a detailed analysis of job competencies for the demand side of talent. The fourth section discusses the analysis of the discrepancy in supply and demand between the two primary industry and education bodies, and the optimisation of talent cultivation. The final section presents the conclusions of this study. This study’s primary contribution lies in its development of a novel, quantitative supply-demand linkage framework that moves beyond prior descriptive or single-perspective analyses, offering actionable insights for curriculum optimization and policy formulation.

The aforementioned policies and national strategies underscore the particular significance of cultivating big data talent within China’s specific socio-economic context. However, the core challenge addressed in this study—the misalignment between academic curricula and the rapidly evolving demands of the data industry—is by no means unique to China. Educational institutions and industries across the globe are grappling with similar issues of skill gaps, curriculum lag, and the pressing need for interdisciplinary integration in data science education [12,16]. While this research is situated within and informed by the Chinese landscape, its primary contribution lies in the development of a methodological framework that integrates talent cultivation side (academic programs) and talent demand side (job market) analyses to bridge this divide. Thus, the findings and the proposed approach offer valuable insights and a replicable model for educators, policymakers, and researchers in other nations facing analogous challenges in preparing a workforce for the digital economy.

## 2. Research framework and methodology

### 2.1. Research framework

**Fig 1:**
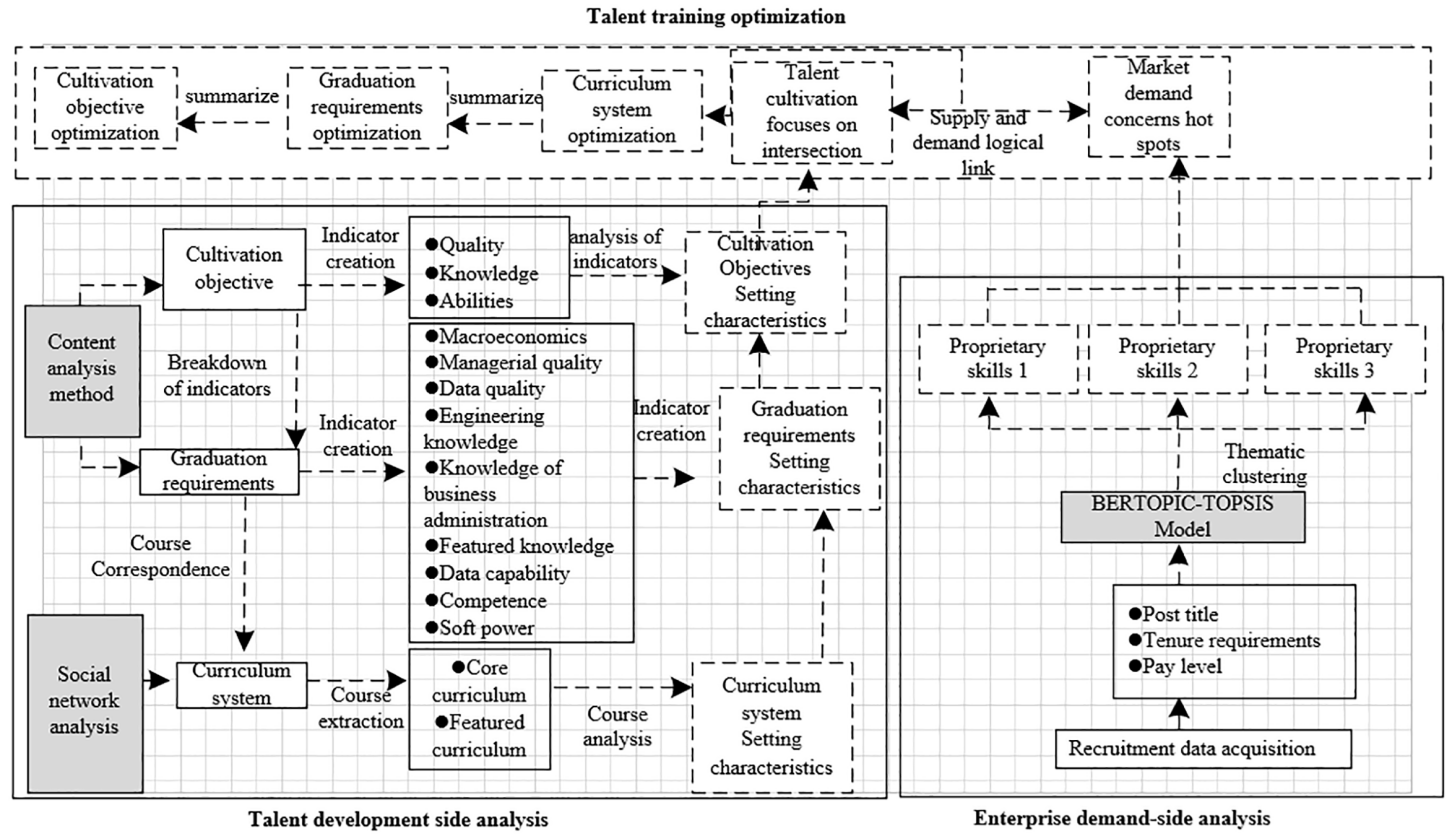
Overall structure of the study.

This study intends to construct a cultivation-demand-feedback cycle cultivation mechanism for big data management and application majors based on the results of text marking on talent cultivation side and talent demand side, and the structure of the study is shown in Fig 1. Firstly, from the perspective of talent cultivation, based on the correlation between cultivation objectives, graduation requirements and curriculum teaching links in the cultivation programs of multiple institutions, this study uses the content analysis method to establish analysis indicators for the cultivation objectives and graduation requirements, and uses the social network analysis method to analyze the curriculum system, so as to find out the characteristics of cultivation and intersection of concerns of various universities in the key links of talent cultivation; secondly, in order to identify the market demand for big data management and application talents, this study uses the text identification results to construct a cultivation-requirement-feedback loop cultivation mechanism. data management and application talent market demand, The BERTopic-TOPSIS model is employed for the purpose of conducting thematic clustering analysis on the network recruitment information pertaining to positions within the field of big data. As a result of this analysis, the specific skills that are required for the performance of different big data positions are identified.; finally, based on the analysis results of the previous section, the talent supply and demand logic link is constructed between supply and demand, and the objective differences between supply and demand of talents are then derived, and the big data curriculum system is optimized in a targeted manner. Data management and application specialty’s curriculum system, graduation requirements, and training objectives.

### 2.2. Research methodology

This study divides the dataset into university-side text data and enterprise-side text data and selects methods based on their feature differences [18].

#### 2.2.1. *Selection of talent-cultivation-demand-side data analysis methods.*

In this study, we collected the training programs of undergraduate majors in big data management and application through official website query, telephone consultation, and email consultation, and a total of 85 programs have been collected, which are representative of different regions, levels, and types of colleges and universities (see Section 3.1 for details).

At present, the Ministry of Education has issued numerous documents related to the construction of undergraduate specialities in universities, which means that at the undergraduate level, China has gradually formed a more complete education system, and the data on undergraduate cultivation programmes collected in this study are precisely formulated by universities under the planning documents, so they have a high degree of homogeneity characteristics. As a scientific method for studying social reality, content analysis is an important method for analyzing structured texts such as educational policies [19] and training programs [19,20]. Its specific analyzing process is executed according to the following steps:(1) Formulation of research questions or hypotheses;(2) Determine the research sample;(3) Establishing the unit of analysis; (4) Indicator construction, coding and elemental entry identification; (5) Interpretation and testing, amount of textual data through a systematic process of interpretation [21], to achieve effective inferences about the reproducibility of the meaning of the text.

It should be noted that the curriculum system’s concise descriptions contain rich cross-disciplinary knowledge, requiring effective analytical methods, and scholars used the social network analysis method to study the intersection of disciplines [22], and found the interrelationships between different disciplines [23]. Therefore, based on the methodological characteristics of social network analysis, this study introduces this method to analyse the professional curriculum system of big data management and application.

#### 2.2.2. *Selection of talent-demand-side data analysis methods.*

In this study, the decision was taken to collect job postings from the 51job and Zhaopin job sites. The search keywords comprised terms related to data analysis, data mining, machine learning, data products, data management, data governance, software development and big data development. The crawler structure was employed in conjunction with manual collection for the purpose of enterprise-side dataset collection (for details, please refer to section 4.1).

At present, although a number of mainstream job boards have formulated occupational classification standards, but in fact the recruitment of talent information developed by the enterprises themselves, the classification standards developed by different job boards are very different, the network recruitment data has obvious differential characteristics, and they are all short text data. Traditional topic models LDA, CTM, etc. have achieved better results in text processing analysis, compared with traditional topic models, the deep learning model BERTopic [24] makes up for the incompatibility between density-based clustering and center-based sampling. BERTopic enables a semantic representation of each term, thereby alleviating vocabulary mismatch issues and illuminating changes in distribution by topic over time [25], The model can well represent the embedded vocabulary and paragraphs in the text, and establish anchors between the resultant topics and the sample literature for the researcher to track and parse, so as to obtain accurate and highly explanatory subject content [26], which is now widely used in the fields of library intelligence, economics, sociology, etc. BERTopic can get post keywords and probability values after clustering the topics, However, the keyword probability value is an important basis for mining the knowledge and ability information in the text of job requirements, but cannot be used as the only criterion. For example, for data development engineers, the use of data development tools such as Spark, Hadoop, etc. is the basic technical skills of the practitioner, however, mastering the basic technical tools is difficult to prove the core competitiveness of the practitioner, and it is necessary to consider the inclusion of other measurement standards. Salary levels reflect the value enterprises place on specific knowledge or skills. The TOPSIS algorithm can be used to synthesize the keyword probability values. Therefore, this study will use the BERTopic-TOPSIS fusion method to extract and analyze the talent demand side data.

BERTopic uses the pre-training model Sentence-BERT [27] to transform each word in the document into a high-dimensional vector representation, preserving the semantic relationships between the sentences; the transformed high-dimensional vectors are mapped into a two-dimensional space using the UMAP algorithm, which better preserves the global and local features of the high-dimensional vectors; the mapped vectors are clustered using the hierarchical density-based HDBCAN algorithm, after which unique clustering labels are generated. The c-TF-IDF algorithm is used to identify and extract topic features and keywords from the different clusters. The clusters with different densities are obtained, and different cluster labels are obtained; based on the c-TF-IDF algorithm, the different clusters are subject feature identification and keyword extraction, and different subjects are obtained. In TOPSIS, the alternatives are ranked according to their distances from positive and negative ideal solutions [28].

The BERTopic model yields keyword probability values that signify their prevalence within a topic cluster. However, prevalence alone is an insufficient metric for assessing the critical importance of a skill. For instance, for a Data Development Engineer, mastery of tools like Hadoop or Spark is a common baseline requirement; however, proficiency in these common tools may not distinguish core competitiveness. Salary level serves as a direct proxy for the economic value that the market assigns to specific knowledge or skills. The TOPSIS algorithm was therefore selected as the optimal method because it enables a multi-criteria decision-making approach. It synthesizes both the prevalence of a skill (derived from the BERTopic probability value) and its economic value (proxied by salary data) into a single composite score.This approach prevents the over-emphasis of frequently mentioned but low-value competencies and ensures that less common yet high-value skills are appropriately weighted in the final ranking. Thus, TOPSIS provides a more holistic and accurate measure of skill importance for talent cultivation guidance than methods relying on a single dimension, such as frequency ranking alone.

## 3. Talent cultivation mode commonality mining

### 3.1. Talent cultivation side data acquisition and preprocessing

The talent development programme contains all the important components of talent development and is an important basis for instructional design [29]. The text of the training programme generally includes the expression of training goals, the expression of graduation requirements, and the setting of the curriculum system and other important teaching links. In this study, the training programmes of undergraduate majors in big data management and application were collated through the official website, telephone consultation, e-mail consultation and other means. As shown in Table 1, the collection targets include universities from different regions, levels and types, which are representative to some extent. The 85 training programmes in question encompass 85 training objectives, 35 graduation requirements and 38 curriculum systems. After the raw data had been organised and analysed qualitatively, data on training programmes with short texts were removed. This was because the text data were too brief to reflect the specific details of the training programme. The training objectives of 63 universities, graduation requirements of 32 universities and the curriculum systems of 38 universities with a total of 1, 090 courses are finally identified as the objects of analysis. After the raw data had been organised and analysed qualitatively, data on training programmes with short texts—defined as those lacking detailed descriptions of specific course content, learning outcomes, or elaborated competency goals—were removed. This was because the text data were too brief to reflect the specific details of the training programme.

**Table 1:** Overview of the collection of training programs.

City Name	Typical Institutions	Nubmer Of Units
Eastern China	Hefei University of Technology, Hohai University, Nanjing Forestry University, Jinan University, Jiangnan University, Nanjing University of Finance and Economics, Weifang Medical College, Shanghai University of Applied Sciences, etc.	18
Northern China	Peking University, Central University of Finance and Economics, Beijing University of Posts and Telecommunications, Beijing University of Traditional Chinese Medicine, The following are some of the most popular universities in China: Capital Normal University, Tianjin Polytechnic University, Tianjin Sports Institute, Hebei University of Economics and Trade, and others.	15
Central China	Wuhan University of Technology, Central South University of Economics and Law, Central China Normal University, Hanjiang University, Wuhan Institute of Biological Engineering, Hubei University of Economics, Zhengzhou University of Finance and Economics, etc.	10
South China	Guangdong University of Finance and Economics, Guangdong University of Technology, Southern University of Science and Technology, South China Agricultural University, Guangzhou Neusoft College, Guangzhou Southern College, Yunnan College of Economics and Management, etc.	10
Southwest China	Sichuan Normal University, Chongqing University of Technology, Southwest University of Finance and Economics, and Chengdu Neusoft University, Chengdu Jincheng College, Jili College, Panzhihua College, Rongzhi College of Chongqing Business University, etc.	9
Northwest China	Xi’ an Jiaotong University, Chang’ an University, Shaanxi Normal University, Xi’ an University of Architecture and Technology, Xi’ an University of Technology, Shaanxi International Business College, Xi’ an Siyuan College, Xijing College, etc.	10
Northeast China	Jilin University, Dalian University of Technology, Harbin Institute of Technology, Shenyang University, Northeast University of Finance and Economics, Dalian University of Foreign Languages, Shenyang City College, Yingkou Institute of Technology	13

### 3.2. Text coding of training programs

The construction of the coding system of the text of the training program is based on two main principles, one is that the cultivation objectives and the teaching links of graduation requirements should reach a supportive relationship [30]; the second is that the construction of the coding system should be based on the three perspectives of quality, knowledge and competence [31]. Based on these two principles, the coding system is constructed according to the following steps:

(1) Establishing indicators for analysing the content of educational goals
  1) By summarising and concluding the training goal texts of 63 universities, three training goal level indicators of quality, knowledge and ability were identified.
  2) The determination of secondary indicators can not only analyse the text of the training objectives of each institution at a finer granularity, but also provide a basis for the establishment of graduation requirement indicators. Some scholars have used the content analysis method to classify the second-level indicators of training objectives of big data management and application specialties [5]. In this study, based on the existing research and the 12 first-level indicators of graduation requirements in the General Standard for Accreditation of Engineering Education, we finally establish 9 second-level indicators of training objectives, as shown in Table 2.
(2) Identification of indicators for content analysis of graduation requirements
  1) In order to effectively decompose the graduation requirements to the training objectives, it is necessary to transform the nine secondary indicators of the training objectives into nine primary indicators of graduation requirements as the base point.
  2) At present, there is little research on the graduation requirement text of big data management and application majors, but scholars have explored the graduation requirement text and curriculum system of related majors by using content analysis methods, such as decomposing the graduation requirement of security engineering majors under the background of new engineering disciplines with fine-grained indicators [32], and analysing the core curriculum of journalism majors under the background of new liberal arts disciplines [20]. This study combines the above results with the breakdown of graduation requirement indicators of the China Association for Accreditation of Engineering Education Programs (CAE2PE) [30], and establishes a total of 46 graduation requirement secondary indicators, as shown in Table 3. The distinction between conceptually close secondary indicators, such as Managerial quality and Management thinking, is defined as follows: Managerial quality refers to inherent traits and ethos (e.g., “possess modern management concepts”), while Management thinking refers to the application of systematic and analytical thought processes to solve management problems (e.g., “apply systems thinking to optimize business processes”). To illustrate the operationalization of the coding process, representative excerpts from the raw text were mapped onto the predefined indicators. For instance, the training objective statement “Cultivate students’ advanced management ideas and high management quality; possess systematic management thinking” was assigned to the primary indicator Inner quality and the secondary indicator Managerial quality. Similarly, the graduation requirement “Be able to design and implement a data warehouse” was coded under the primary indicator Data development application capacity and the secondary indicator Data governance and management. This systematic coding approach, grounded in the established quality–knowledge–competence framework and aligned with CAE2PE standards, ensured a consistent and replicable annotation process across all textual materials.

**Table 2:** Indicators for analyzing training objectives.

Level 1 indicators	Secondary indicators	Example of correspondence for secondary indicators
Inner quality	macro quality	To develop students’ creative thinking; to have a sense of social responsibility;
	managerial quality	Possess modern management concepts; advanced management ideas and high management quality; systematic management thinking;
	data quality	Understand the life cycle of data in the networked information society; understand the dynamics of big data technologies and their industry development;
Knowledge-related	economic knowledge	Good theoretical foundation in economics and management; ability to understand and apply basic accounting and finance theories;
	engineering knowledge	Master the theory of efficient collection, processing and management of data sets; Systematic mastery of mathematics and statistics; good foundation in mathematics and science;
	featured knowledge	Cross-fertilization of data science and Chinese medicine; background in kinesiology;
Abilities	soft power	Strong big data management and big data technology application skills; big data application system design, development and management;
	competence	Business process optimization; operation and maintenance management and decision-making consulting; realize business operation analysis and enterprise management decision-making support;
	data capability	Have some international communication skills; have the ability to express themselves in language and writing; have the ability to communicate, coordinate and make decisions;

**Table 3:** Indicators of graduation requirements and curriculum system analysis.

Level 1 indicators	Secondary indicators
Macroeconomics	thoughts and perspectives
	law and ethics
	political literacy
	research spirit
	wellness
	art and culture
Data quality	data legal ethics
	data frontiers
	data-driven thinking
Managerial quality	innovative management
	business law and ethics
	management thinking
Engineering knowledge	basic computer knowledge
	mathematical statistics
	data acquisition and processing
	data storage management
	data mining analysis
	data visualization
	data security
	natural sciences
Economic knowledge	foundation of economics and management
	financial services
	e-commerce
	engineering management
	logistics
	marketing
Subject knowledge	engineering
	sociological
	study of medicine
Data development application capacity	big data analytics
	data governance and management
	information management
	system development
	data science R&D
Competence	management decision
	operations management
	project management
	business process
	marketing program
Soft power	innovation capacity
	work independently
	communication skills
	practical ability
	written expression
	teamwork
	lifelong learning

### 3.3. Core and specialty course extraction

Cultivating talents in big data management and application is not a simple combination of knowledge in two disciplines, namely big data and management, but rather emphasizes the theoretical and practical innovations that come from the intersection of big data technology and multidisciplinary knowledge. Therefore, in order to further understand the current status of curriculum integration in this specialty, the following section will use social network analysis to dig out the representative core courses as well as the characteristic courses of 38 institutions, so as to deeply analyse the current status of curriculum integration in the context of multiple disciplines.

**Fig 2:**
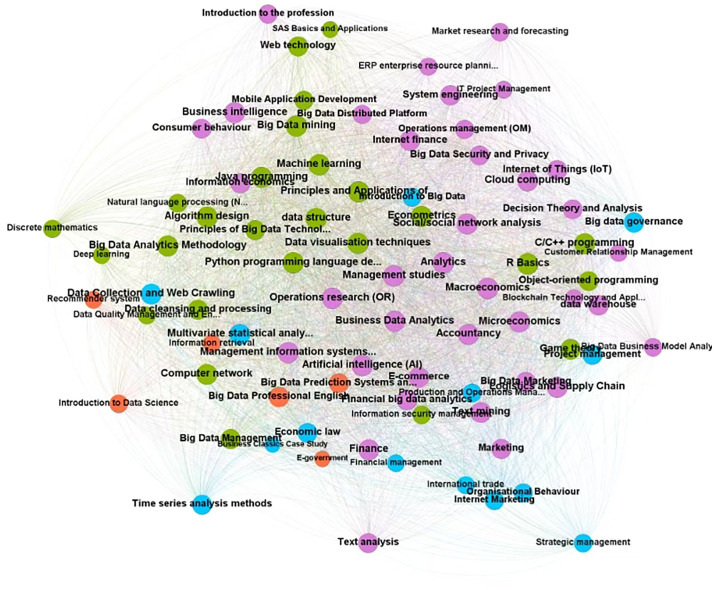
Curriculum network.

Using social network analysis to identify the core courses, we first classified and counted the courses according to their titles, merged the courses with similar expressions, such as “Data Structures” and “Data Structures and Algorithms” into “Data Structures” and deleting the general fundamentals courses. And then constructed a network of course-curriculum relationships using a VBA program and applied centrality indicators to identify the core courses. Finally, 1090 courses are merged into 349 courses, and the course network relationship is constructed by Gephi 0.10.1 software, and the network relationship is shown in Fig 2, where the nodes in the network represent the courses offered, and the larger the node and the closer the course is to the centre of the course network, the more courses are offered by more collecting institutions. The core courses of the big data management and application programme should mainly include management knowledge and knowledge related to data technology, and have formed the following courses: “Analytics”, “Management Science”, “Database System Principles and Applications”. It has formed the group of professional basic courses with “Operations Research”, “Management Science”, “Database System Principles and Applications” and “Big Data Mining” as the core, and the group of professional basic courses with “Logistics Big Data Analysis”, “Medical Big Data Management” and “Healthcare Big Data Management” as the representatives. It also has a number of industry application course groups, including “Logistics Big Data Analysis”, “Healthcare Big Data Management” and “Energy Big Data Analysis”, which have certain cross-disciplinary characteristics.

Fig 2 shows many courses, the core curriculum can be further refined and extracted to be measured using social network degree centrality metrics. Degree centrality is regarded as one of the fundamental metrics in social network analysis, employed extensively across diverse research domains to assess the positions of actors in relation to their immediate neighbours and the connections they have [33]. The identification of core and specialty courses was achieved through social network analysis (SNA). A course co-occurrence network was constructed based on the frequency of courses offered across the collected curricula. Degree centrality was selected as the primary centrality measure for this analysis, as it directly reflects the number of direct connections a course has within the network, effectively identifying which courses are most commonly shared across diverse programs and thus represent the foundational knowledge core. To objectively determine the threshold for designating a course as ‘core’, the calculated degree centrality values for all courses were ranked in descending order. A clear cutoff was established by selecting the top 30 courses based on the highest degree centrality values. This threshold effectively captured the most central and commonly required courses within the network, as illustrated in Table 4. Courses that were highly specific to only a few institutions’ unique disciplines (e.g., “Medical Big Data Management”) emerged as peripheral nodes in the network and were thus identified as characteristic specialty courses. From Table 4, included in the core curriculum of the program are 11 basic courses in economics and management (Management, operations research, Microeconomics, etc.), 8 basic courses in computer science (Principles and applications of database systems, Data structures, Python programming language design, etc.), and 11 basic courses in data science (Statistics, Multivariate statistical analysis, Principles of big data techniques, Data visualization techniques, etc.), explaining that the program is primarily based on knowledge of economic management disciplines, with a core of expertise in data science and computer science disciplines.

**Table 4:** Extraction results of core courses.

Rankings	Course Name	Kilowatt-hour one’ s nature	Rankings	Course Name	Kilowatt-hour one’ s nature
1	Management studies	12. 63	16	C/C++ language programming	6. 34
2	Principles and applications of database systems	11. 83	17	Java programming	6. 14
3	Data structure	11. 43	18	E-commerce	5. 93
4	Operations research	10. 38	19	Business data analytics	5. 59
5	Analytics	10. 20	20	Multivariate Statistical analysis	5. 32
6	Python programming language design	10. 06	21	Social/Social network analysis	5. 226
7	Macroeconomics	9. 73	22	R basics	5. 119
8	Big data mining	9. 53	23	Computer network	5. 029
9	Microeconomics	8. 06	24	Principles of big data technology	4. 912
10	Management information systems and development	7. 96	25	Business intelligence	4. 688
11	Data visualization techniques	7. 73	26	Big data analytics methodology	4. 679
12	Accountancy	7. 50	27	Algorithm design	4. 664
13	Machine learning	6. 92	28	Text analysis	4. 567
14	Artificial intelligence	6. 56	29	Data cleansing and processing	4. 553
15	Econometrics	6. 43	30	Logistics and supply chain	4. 328

### 3.4. Current situation and problems on the talent training side

Through the coding of the text of cultivation goals and graduation requirements and the in-depth excavation of core courses and special courses in the curriculum system, this part will conduct frequency statistics of the nodes in Tables 2 and 3, and combine the extracted core courses and special courses to make a comprehensive analysis. In this part, the frequency statistics of the nodes in Tables 2 and 3 will be carried out, and the analysis will be carried out comprehensively with the extracted core courses and special courses, and finally the conclusion of talent cultivation will be drawn based on the two perspectives of cultivation focus and cultivation problems..

#### 3.4.1. *Characterization of training objectives.*

(1) A core competency of the programme is the ability to apply data.

**Fig 3:**
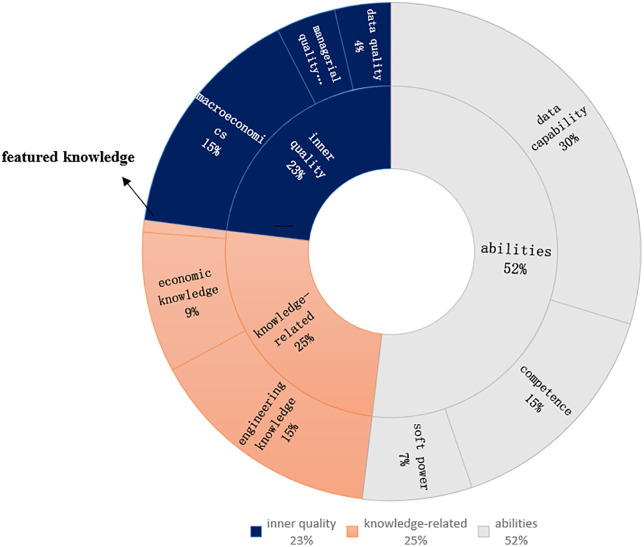
Results of the analysis of indicators of training objectives.

The results of the proportion of each indicator of educational goals are shown in Fig 3, where quality refers to the training of students’ psychology and thinking in the process of teaching, ability refers to the comprehensive practical ability that should be formed by students to engage in a certain field of work, and knowledge refers to the theories and methods that must be mastered by students to carry on thinking and ability [34]. From the perspective of the first-level indicators, the proportion of quality and knowledge indicators is 23% and 25% respectively, and the proportion of ability indicators is 52%, which shows that, overall, universities pay more attention to improving students’ practical aspects and application ability. Further observation of the second-level indicators from the perspective of competence shows that the proportion of data competence indicators is 30%, which is significantly higher than that of economic management competence indicators, indicating that the programme focuses more on enabling students to solve the problems at the level of big data application in practice, rather than management competence or computer technology and development competence from the perspective of a single discipline.

(2) There is a general lack of effort made to help students become professionalised.

As shown in Fig 3, the proportion of macro quality indicators is the highest, which is due to the fact that the cultivation objectives need to meet the macro quality requirements of the Ministry of Education for undergraduate education, but the proportion of the quality indicators of economics and management (4%) and the proportion of the quality indicators of data (4%) are much lower than the proportion of macro quality indicators (15%). The percentage of indicators in Fig 3 reveals the insufficiency of universities in the cultivation of professionalism. Ideas are the forerunner of practical application, and quality requirements can actually lead students to pursue knowledge and skills at a higher level. Therefore, universities should strengthen the cultivation of students’ economic and management qualities and data qualities while cultivating students’ macroscopic qualities, so as to enable students to form the professional qualities of big data management and application, and ultimately guide students to form the ability of big data application.

#### 3.4.2. *Characterization of graduation requirements.*

(1) Universities generally emphasize data science foundations and background in economic and management knowledge

**Fig 4:**
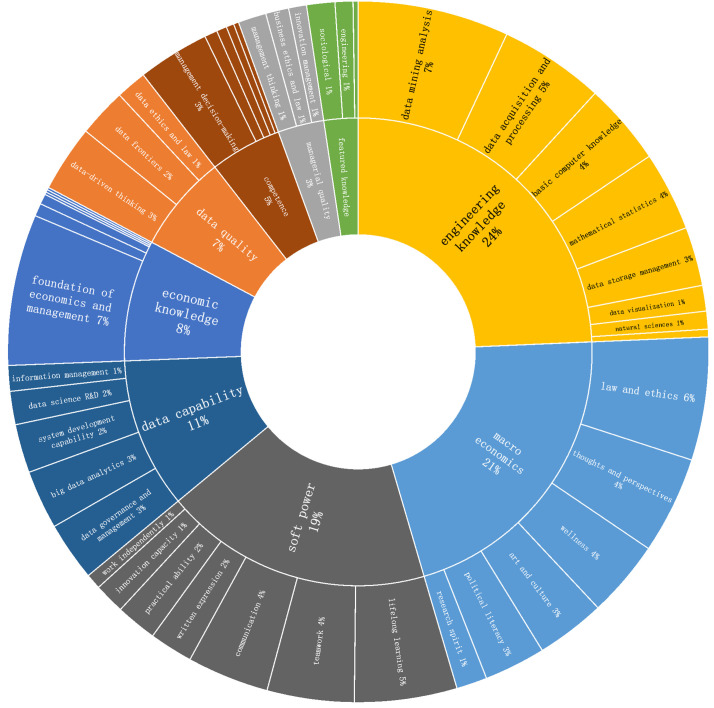
Results of the analysis of graduation requirement indicators.

The percentage of each indicator of graduation requirements is shown in Fig 4. From the perspective of first-level indicators, in addition to the macro qualities and soft skills specified by the Ministry of Education, the proportion of engineering knowledge indicators is 24%, and the proportion of economic and management knowledge indicators is 8%, both of which are more prominent, which verifies the professional characteristics of this specialty of cross-fertilisation of big data technology and economic and management knowledge. From the viewpoint of the second-level indicators of engineering knowledge, the proportion of data mining and analysis indicators is 6.97%, which is at a high level, and the proportion of matching knowledge indicators of computer underlying knowledge, data collection and processing, data storage and management is also at a high level. From the viewpoint of second-level indicators of economic and management knowledge, the proportion of indicators of basic knowledge of economics and management is 6.97%, which is at a high level, while there are fewer expressions of segmented knowledge of finance and finance and other management fields. The results of the above indicator weighting show that most institutions train basic economic and management knowledge, such as economics and management, as the background knowledge of the discipline, while the knowledge base of big data analysis and mining is regarded as the knowledge core of the discipline.

(2) The profession has not yet developed a systematic paradigm for the development of data application skills

Although the cultivation goal-setting process attaches great importance to big data application capabilities, from the perspective of the first-level index of graduation requirements, the proportion of the data capabilities index (11%) is significantly lower than that of the cultivation goal-setting process (30%). This phenomenon mainly shows the problem of ambiguous results in the development of big data application ability, which is mainly evident in the following aspects: the majority of universities define data ability as “strong big data management and big data technology application ability” in their cultivation goals. This is an abstract and vague concept that lacks clear direction. The formulation of graduation requirements should be in line with and operationalise the abstract cultivation goals. The graduation requirements should be formulated in a precise manner to reflect the desired big data application capabilities. Graduation requirements should be formulated in such a way that the more abstract cultivation objectives can be crystallised into concrete expressions, such as ‘being able to complete financial risk and credit analysis’ and ‘being familiar with technical standards in the field of data science’, etc. However, the results of the analysis show that most of the institutions’ graduation requirements lack detailed expressions of the skills that students should develop. However, the results of the analysis show that most institutions’ graduation requirements lack detailed expressions of the skills that students should develop.

This point is strengthened by contrasting it with the practices of institutions that successfully operationalize these goals. For instance, Peking University’s program, as identified in our curriculum network analysis, offers specialized courses in Intelligence Big Data, which implies a curriculum designed to translate abstract application abilities into concrete domain-specific competencies. Similarly, Hefei University of Technology distinguishes itself with courses in Logistics Big Data Analysis, demonstrating a clear pathway to applying data skills within a specific industrial context (refer to Section 3.3 and Fig 2 for course network details). These examples, evident within our sample, illustrate how vague goals can be operationalized into measurable skills through specialized, application-oriented coursework.

#### 3.4.3. *Characterization of the curriculum.*

(1) Specialized core curriculum showing hierarchical and modular characteristics

As shown in Table 4, computer science courses and data science courses show a hierarchical character, which is mainly manifested in the following: Programming courses such as Python and Java serve as the technical foundation, while courses such as Data structure, Computer network, Database, Data mining, etc. serve as the knowledge foundation, and the integration of technical and knowledge courses can enable students to form top-level competencies such as Business data analysis, Management information system development, Artificial intelligence, etc. can enable students to form top-level competencies such as Business data analysis, Management information system development, and Artificial intelligence. The modularisation characteristics of the economic management courses are evident in their structure, which incorporates a knowledge base Encompassing management, Operations research, and Economics, among other disciplines. The universities extend the courses of accounting, marketing, Logistics and supply chain, and other management subdivision fields, which provide support for the students’ practical ability of big data management.

(2) The phenomenon of combining traditional specialized courses is serious, and a systematic specialized course has not yet been formed.

Observing the core courses extracted from Table 4, it is not difficult to find that the core courses of this major are a combination of courses from different institutions that simply retain the characteristics of the predecessor majors of big data management and application (Information management and information system), and there is the phenomenon of copying the traditional professional courses of management and computer science. Secondly, although Fig 3 shows a certain scale of characteristic courses of big data, by checking the correspondence between characteristic courses and universities, it is concluded that the characteristic courses are concentrated in a few institutions: for example, intelligence big data courses are concentrated in Peking University, medicine big data courses are concentrated in Beijing University of Traditional Chinese Medicine, and more institutions do not combine their own disciplines to set up specialization courses. More universities do not combine their own disciplines to set up specialty courses, which makes it difficult to form a cultivation paradigm of multidisciplinary cross-convergence and multitechnology cross-border fusion.

## 4. Talent demand side job capacity mining analysis

Universities, as an important force in cultivating talents, should develop a reasonable talent cultivation programme based on social demand to cultivate more core talents needed by the country and society. Based on social demand, this part will obtain key job information of big data-related jobs from domestic mainstream recruitment websites, such as job title, job requirements, salary, etc., and after data preprocessing and BERTopic model training, extract the knowledge and ability keywords corresponding to different jobs, and ultimately determine the focus of the talent demand. The job board information collected by the Institute is publicly accessible and has no privacy restrictions.

### 4.1. Talent demand side data collection and pre-processing

The data published on the recruitment website most accurately reflects the market demand for data analytics talent [35]. According to the China Online Recruitment Industry Market Research and Development Report 2022, 51job ranks first in terms of market share in the online recruitment industry, followed by Wisdom Link Recruitment. Therefore, in order to ensure the most accurate representation of the data, this study has chosen to collect recruitment information from the 51job and Zhaopin websites.

To obtain sample data that is representative of the current job market, we collected job postings between September 11, 2022, and September 11, 2024. In the present study, a total of 12,267 job postings were retrieved using a combination of data analytics, data mining, machine learning, data products, data management, data governance, software development and big data development as the search keywords. All of the aforementioned job postings were captured using a crawler structure combined with manual collection. the recruitment information are still problems of inconsistent format and more interfering information, and it is essential to ascertain the quality of the data prior to mining the key information: (1) Unresolvable characters generated by webpage structure problems; (2) Irrelevant information such as “work benefits” and “insurance and vacation”; (3) Unrelated job information, such as “data entry clerk”, “data clerk” and other unrelated jobs. Following the completion of the data cleansing process, a total of 5,349 valid job postings were identified.

The selection of 51job and Zhaopin is justified by their dominant market share in China, ensuring extensive coverage. However, it may introduce bias towards certain industries, company sizes (e.g., Liepin is often associated with mid-to-senior level positions in larger firms), and platform-specific terminologies. This limitation is acknowledged, and findings should be interpreted within this context.

### 4.2. Posting requirements theme mining

This study integrated BERTopic and TOPSIS, with the main steps consisting of the following three:

Step 1: BERTopic topic modelling and keyword extraction
Step 2: TOPSIS input matrix construction
Step 3: TOPSIS composite score calculation

This study uses the deep learning model BERTopic for text topic modelling, aggregating similar job descriptions into skill topics through semantic understanding. The HDBSCAN clustering algorithm is used for topic clustering and c-TF-IDF for topic representation to create dense clusters and extract representative words for each skill topic. Topic consistency is an important indicator of topic quality, so this study introduces it for this purpose. The TOPSIS algorithm was used for comprehensive scoring based on the probability value of job keywords and salary, and the skills were ranked by comparing their proximity to the “ideal important skills”.

#### 4.2.1. *Thematic coherence.*

Topic consistency is a measure of topic interpretability. By taking topic consistency into account, it is possible to avoid both topic overfitting and the generation of meaningless topics. Roder summarized the commonly used methods for calculating topic consistency, and demonstrated that Cv is the best coherence metric [36]. Cv The higher the score, the better the topic coherence. By calculating the distance of words in a topic, the differentiation between topics is inferred, so as to achieve the effect of scoring individual topics, the topic coherence calculation is shown in Equation (1).


$$\begin{array}{c} C_{v}=\sum {\mathrm{\backslash nolimits}}_{\text{2}}^{M}\;\sum {\mathrm{\backslash nolimits}}_{l=\text{1}}^{m}log\frac{D\left(\nu_{m}^{k},\nu_{l}^{k}\right)+\text{1}}{D(v_{l}^{k})} \end{array}$$ (1)

Cvis the consistency-distinguishing measure validity, and $v^{k}=(v^{\text{1}},\ldots \;,\nu_{m}^{k})$ denotes the list of M words in topic K. D(ν) is the number of job postings containing the word ν of job postings, and $\;D(\nu ,\;\nu^{\prime })$ is the number of words ν is the number of job postings that contain the word $\nu^{\prime }$ the number of job posting entries that co-occur at least once. Higher thematic coherence indicates better thematic coherence and higher thematic interpretability.

#### 4.2.2. *BERTopic model topic clustering.*

**Fig 5:**
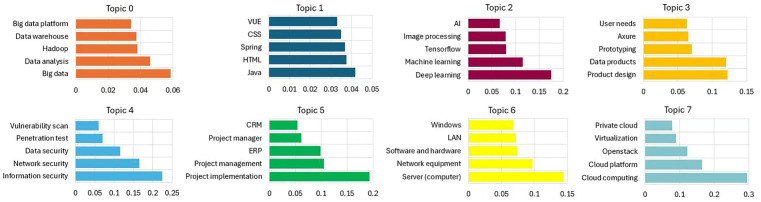
Distribution of feature words of big data industry job recruitment text study themes (some themes).

In this study, BERTopic is used for theme clustering analysis, the pre-processed recruitment text is input into the BERTopic model, the BERTopic model employs a pre-trained model to transform the text into embedded vectors. Subsequently, UMAP is utilised to diminish the vector dimensionality and optimise the clustering process, after the dimensionality reduction is completed, the clustering is carried out using HDBSCAN, and finally, the TF-IDF algorithm based on the category is used for theme extraction, some theme feature words are shown in Fig 5.

#### 4.2.3. *Thematic consistency test.*

**Fig 6:**
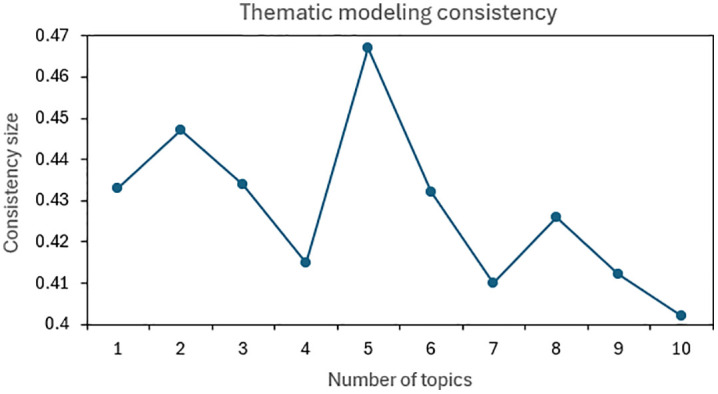
Thematic coherence.

Cluster analysis for recruitment text, and extract the first 50 keywords of each theme for theme consistency index calculation, as shown in Fig 6, when the number of themes is 5 shows that the highest theme consistency index value, the theme extraction quality is the best. Combined with the results of the theme consistency test, the five themes with the highest significance of the BERTopic model training output results are selected. Combining the competency requirements of Big Data industry jobs and the relevant descriptions in the IT career graph, the five themes can be named “Data Development Engineer”, “Data Scientist”, “Big Data Product Manager”, “Data Security Assessment Engineer”, “Data Implementation Engineer”.

#### 4.2.4. *TOPSIS composite score.*

For enterprises, talents with solid basic ability and outstanding core ability are worth offering higher salary level. Keywords can be used as an important basis for enterprises to recruit talents after calculating the realisation value, in order to ensure that both the frequency value and the important basis are included in the importance ranking of keywords, this study uses the TOPSIS algorithm to comprehensively score the frequency and salary.

The algorithm for keyword realization based on salary level is first introduced [37], which is calculated in Equation (2).


$${Value}_{i}=\frac{\sum_{j=\text{1}}^{m}s_{j}*w_{ij}*\frac{\text{1}}{n_{j}}}{\sum_{j=\text{1}}^{m}w_{ij}}$$ (2)

Where ${Value}_{i}$ denotes the value of the first i realization value of the first keyword, and $w_{ij}$ denotes the value of the firsti whether the keyword appears in the first j job posting, which takes the value of 0 or 1, and m is the total number ofrecruitment, and${\;s}_{j}$ is the salary of j position, and $n_{j}$ is the total number of keywords in recruitment.

The TOPSIS algorithm must first determine the maximum score for the keyword frequency and keyword realisation value metrics ($D_{i}^{+}$) and the minimum score ($D_{i}^{-}$), The distance between each evaluation programme and $D_{i}^{+}$, was then determined using the Euclidean distance formula. Then the distance between each evaluation programme and $\;D_{i}^{+}$, $D_{i}^{-}$ was then calculated using the Euclidean distance formula. The distance between each evaluation program and finally calculate the composite score $G_{i}$, the calculation formula is shown in Equations (3), (4), (5). The results of the keyword composite score calculation will be available in the Table 5.

**Table 5:** Value of the composite keyword score.

Thematic	Keyword examples and realisation value
Data Development Engineer	data analytics (0.83); java (0.658); database (0.614); data warehouse (0.543); development experience (0.5); sql (0.5); hadoop (0.421); development engineer (0.41); etl (0.393); mysql (0.389).
Data Scientist	deep learning (0.842); machine learning (0.696); tensorflow (0.496); image processing (0.482); artificial intelligence (0.427); python (0.421); computer vision (0.396); target detection (0.34); machine learning algorithms (0.323); pattern recognition (0.32)
Big Data Product Manager	data services(0.86); product design (0.65); prototyping (0.386); user requirements (0.32); axure (0.307); solutions (0.292); requirements analysis (0.288); data analysis (0.252); product development (0.245); logical thinking (0.234)
Data Security Assessment Engineer	information security (0.914); network security (0.589); data security (0.577); penetration testing (0.527); vulnerability scanning (0.446); security incidents (0.437); web security (0.413); system security (0.35); firewall (0.317); cisp (0.307);flink (0.301); data analytics (0.264); Python (0.243); Java (0.233)
Data Implementation Engineer	project execution(0759); project management (0.743); erp (0.597); project manager (0.523); crm (0.489); solutions (0.482); erp implementation (0.462); customer service (0.459); business process (0.361); requirements analysis (0.357)

Note: Due to space constraints, Table 5 only shows the top 10 keywords in terms of composite score.


$$D_{i}^{+}=\sqrt{\sum_{j=\text{1}}^{\text{4}}{{(y}_{i\dot{J}}-y_{ij}^{\text{max}})}^{\text{2}}}$$ (3)

$$D_{i}^{-}=\sqrt{\sum_{j=\text{1}}^{\text{4}}{{(y}_{i\dot{J}}-y_{ij}^{\text{min}})}^{\text{2}}}$$ (4)

$$G_{i}=\frac{D_{i}^{-}}{\left(D_{i}^{-}+D_{i}^{+}\right)}$$ (5)

The results in Table 5 show that technical tools such as “hadoop” (rated 0.421) should be included more extensively in the curriculum, while soft skills such as “logical thinking” (rated 0.234) should be supplemented with project work.

### 4.3. Conclusion of industry demand side analysis

#### 4.3.1. *Data development engineer.*

Data Development Engineer is primarily tasked with the optimisation of big data product architectures, the development of technical solutions and the development of big data software applications [38]. The position is primarily concerned with the integration of software engineering disciplines and big data processing and analysis technologies, which places high demands on the underlying computer technology and data science knowledge. At the knowledge level, data storage and retrieval, distributed system architecture, data processing, the ability to analyse and mine data are the basic skills required for this position; at the language level, Java, Python, SQL are the mainstream languages to be mastered for this position; from the perspective of tools and technologies, ETL, Hive, Hadoop, Spark, Flink and other mathematical management or distributed data processing tools and technologies are the core requirements of the position; from the perspective of final ability, the position is expected to be able to form the basic ability of both big data platform design and big data software development.

#### 4.3.2. *Data scientist.*

Data scientists apply algorithmic knowledge to transform real-world problems into data problems, then use data science theories and tools to derive insights from data [8]. From Table 5, it is clear that this position requires a certain level of expertise in the fields of data science and artificial intelligence, with particular emphasis on a comprehensive understanding of machine learning, deep learning theories, and related concepts. From a theoretical perspective, knowledge of machine learning algorithms and deep learning is in high demand by recruiters in this field, and mastering this knowledge requires a solid foundation in mathematical theories and algorithms. From a technical application perspective, the use of Python language, Tensorflow framework, and AI-related technologies are basic requirements for engaging in the data science industry. From an ultimate application capability perspective, the position requires practitioners to form a systematic machine learning system. The position requires the practitioner to form a systematic machine learning, deep learning, and other capabilities and be able to apply them to the field of artificial intelligence.

#### 4.3.3. *Big data product manager.*

Big Data Product Manager is responsible for Big Data product requirements, planning, design, delivery, and continuous iterative product optimization [38]. Table 5 shows that this position requires strong expertise in data products, including product design and requirements analysis, focusing on the combination of product design knowledge and computer-related technology to form data product management capabilities. From the perspective of knowledge, it is necessary to master the knowledge related to data products, product design and demand analysis; from the perspective of technical application, the position has high requirements for Axure application, data analysis and product development.

#### 4.3.4. *Data security assessment engineer.*

Data Security Assessment Engineer has the ability to assess big data security and analyze big data security issues, and be able to prepare the corresponding documents [31], the position needs to have a solid grasp of network security knowledge, data science knowledge, the ability to apply software testing knowledge in the context of developing and implementing big data security technology programs is essential. Combined with Table 5, from the perspective of knowledge, the position has high requirements for information security, network security, information security knowledge, from the perspective of technical skills, the position needs to be familiar with penetration testing, vulnerability scanning, security incidents, CSIP, Java and other technologies for practical use.

#### 4.3.5. *Data implementation engineer.*

Data Implementation Engineer is responsible for the deployment, implementation, and follow-up of big data project site [39]. Combined with Table 5, from the perspective of knowledge, this position has high requirements for project implementation, project management, and other knowledge, and needs to understand the main stages of big data project implementation. From the perspective of technical skills, this position needs to have a certain understanding of ERP and CRM-related technical knowledge. From the perspective of the final ability to be formed, it should have a strong ability to implement projects under typical big data business scenarios, furthermore, the ability to utilise appropriate big data project implementation standards is essential to facilitate customer access to data product deployment and online services, in addition, it is also necessary to be able to complete the preparation of the project requirements specification and acceptance report and other documents.

## 5. Analysis of the Difference between Supply And Demand and Optimization of Talent Cultivation in the Dual Subjects of Industry and Education

### 5.1. Principles of building links corresponding to the supply and demand of talents

In the context of deep integration of talent cultivation and industrial demand development, China’s colleges and universities are relatively lagging behind in the recognition of big data talent knowledge demand update, talent structure adjustment and skill demand iteration, and the demand for industrial skills and competence characteristics is still at the theoretical assumption and a priori stage, and there is a lack of strong supporting evidence at the practical level of excavating the characteristics of talent demand and feedback on talent cultivation [40]. Therefore, in order to solve the information asymmetry problem faced by both industry and education, this part will construct the logic link between talent supply and demand based on the analysis results of the talent cultivation side and the industry demand side in the previous section, with the following construction principles:

Principles for building key links in talent cultivation. The relationship between training objectives and graduation requirements is determined by the statistical frequency of content analysis. For instance, if the statistical frequency of the ‘management thinking’ node in graduation requirements is 11, the relationship strength between the ‘management thinking’ node and the ‘economic and managerial quality’ node in training objectives is also 11. The relationship strength under different types of indicators is derived by using the similarity of texts. For instance, if the statistical frequency of the ‘management thinking’ index node in the graduation requirements is 11, then the relationship strength between the ‘management thinking’ node and the ‘economic and managerial quality’ index node of the cultivation objectives is also 11. The relationship strength under different types of indexes is derived by using the text similarity. For instance, the similarity between the text describing ‘macro quality’ in the training objectives and the text describing ‘data mining analysis’ in the graduation requirements is 7.847, and the strength of the relationship between the two is 7.847. To illustrate this further, if the degree centrality of the core course is 11.83, the strength of the relationship between the ‘data storage management’ indicator in the graduation requirements and the core course ‘database system principles and applications’ is also 11.83. The text similarity formula is shown in Equation (6), which measures the semantic relevance of the training objectives and graduation requirements text through cosine similarity:


$$\text{Similarity}(\text{A},\text{B})=\frac{\sum_{\text{i}=\text{1}}^{\text{n}}{\text{A}}_{\text{i}}*{\text{B}}_{\text{i}}}{\sqrt{\sum_{\text{i}=\text{1}}^{\text{n}}{\text{A}}_{\text{i}}^{\text{2}}}*\sqrt{\sum_{\text{i}=\text{1}}^{\text{n}}{\text{B}}_{\text{i}}^{\text{2}}}}\;$$ (6)

Where *A*_*i*_ and *B*_*i*_ represent the word frequency vectors of text A (e.g., “macro quality”) and text B (e.g., “data mining analysis”), respectively. The similarity value is [0, 10], the larger the value, the stronger the semantic association.

Principles for building the link between supply and demand of talents: (1) When the core courses do not appear in the job requirements, a link should be built between the graduation requirements and the job. For example, if the keyword “communication ability” appears in the post of data development engineer in Table 5, but there is no corresponding core course, then the node of “communication” indicator in the graduation requirements should be linked with the post, and the strength of the linkage should be “ Communication Skills” keyword combined score (0.324). (2) When the keywords of the job requirements appear in the core curriculum, the core curriculum should be linked to the job construction. Such as data development engineer job requirements appear in the “database” keyword, the job and the “database system principles and applications” core courses should be associated with the strength of the association for the “database” keyword score (0.324). The strength of the linkage is the combined score of the keyword “database” (0.543). (3) If there is no core course or post keyword corresponding to the node of “political literacy”, it will not be included in the research scope.

**Fig 7:**
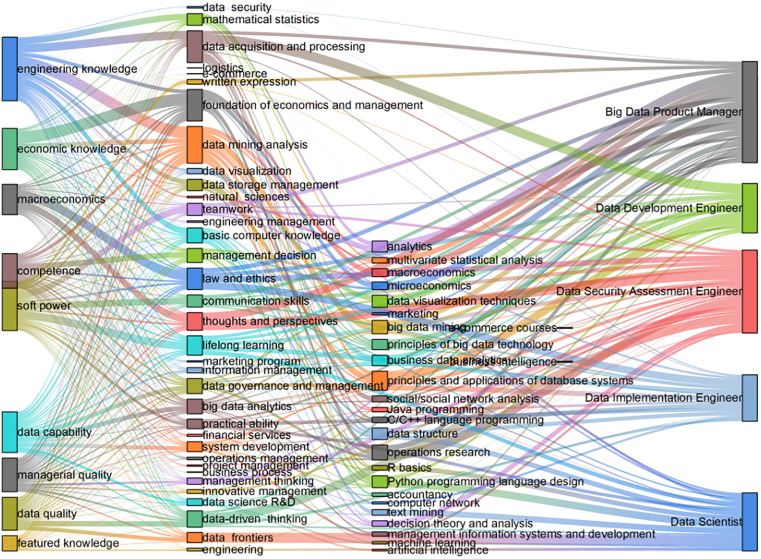
The logical link between the supply of and demand for talent in big data management and applications.

As shown in Fig 7, The size of the node indicates its importance (for example, the “data platform development” node is larger because it is related to high-frequency courses and job requirements), and the thickness of the connecting line reflects the strength of the relationship (for example, the thick line between “data quality” and “data security assessment engineer” indicates a strong correlation). For example, the thick line between “data quality” and “data security assessment engineer” indicates a strong correlation.

### 5.2. Definition and prospect of cultivation direction of big data management and application specialization

#### 5.2.1. *Definition of training directions.*

The direction of talent cultivation should be determined in conjunction with the characteristics of the specialty, and the nature and mission of different types of specialties are quite different. Combined with the nodes of the positions in Fig 7, the nodes of both big data product manager and data security assessment engineer are noticeably large, suggesting that the position in question is highly compatible with the aforementioned specialties and should be the main cultivation direction of professional talents, while the nodes of the positions of data scientist, data development engineer and data implementation engineer are small, indicating that professional talents should master some of the knowledge and skills of the position, but this should not be the main focus of talent cultivation. It is important for professionals to have a certain level of knowledge and expertise in their field, but this should not be the primary goal of talent training. Therefore, big data management and application specialisation can be broadly divided into three training directions, according to the order of professional suitability: data management direction, data analysis direction, and data platform development direction.

#### 5.2.2. *Market demand frontier outlook.*

Through the mining of recruitment text in the previous section, it was concluded that the hot demand of the current big data talent market, but due to the cost and risk of the digital frontier technology for enterprises, it is difficult to convey the cutting-edge and trend-setting nature of big data technology in the context of large-scale enterprise recruitment text extraction results, and the university as an important research and development base of the digital frontier, the training programme should not only meet the current social needs, but also in line with the projected growth trajectory of the big data industry. The development of the big data industry and cutting-edge technologies, such as data analytics, is now a topic of discussion among scholars and organisations, and some scholars have applied graph analytics to the design of intelligent products in the home environment, which can accurately and quickly carry out diverse and dynamic product design [41]. In the field of data governance, data weaving, decision intelligence and other technologies are regarded as cutting-edge technologies of big data management applications, and the new challenges that cutting-edge governance technologies bring to the organisational structure of enterprises have also triggered academic thinking [42].

### 5.3. Recommendations for the optimisation of talent development programmes

Through the above analysis, the cultivation direction and the logical link between the supply and demand of talents in the major of big data management and application have been derived. This part will optimise the curriculum system, graduation requirements and cultivation objectives of the major based on the analysis results, so that the major can provide students with cultivation paths that are more in line with the objective needs and diversified development of talents.

#### 5.3.1. *Recommendations for optimising the curriculum.*

From Fig 7, it can be seen that although there are differences in the knowledge and skills corresponding to different positions, there is also a common intersection. The intersection corresponding to different positions is the knowledge and abilities required for big data management and application professionals, which is reflected in the general knowledge and compulsory courses, while the core courses of the professional direction with clear orientation should be set up for the unique knowledge and abilities of different positions.

The suggestions for optimising general knowledge and compulsory courses are as following. First of all, colleges and universities should start cultivating students’ academic qualities. As an important innovation education platform and professionalism experimentation base, colleges and universities always play a central role in cultivating innovative talents, so colleges and universities should pay attention to cultivating students’ innovative thinking, business thinking and big data thinking, and be able to organically integrate the three to form “Frontiers of Digital Management” and “Frontiers of Digital Technology”, “Frontiers of Digital Technology” and other innovative digital thinking training courses. Second, at the undergraduate level, universities should focus on consolidating the knowledge base in computer science, data science and business and management. As can be seen from Fig 7, different positions have higher requirements for programming, database, data analysis, big data technology, so data structure, Java, Python, higher mathematics, database principles, big data analysis foundation courses should be included in the scope of compulsory courses and combined with management, economics, decision theory and other economic courses.

Taking into account the quantitative mapping of talent supply and demand in Fig 7, the optimisation of the core curriculum for the professional direction is proposed as follows:

For the direction of data management, the courses at the level of technical methods should include courses on data acquisition, processing, storage, analysis, visualisation, security protection, etc.; the courses at the level of management should include courses on skills such as data quality management, master data, metadata management, etc.; through the organic fusion of data technology and management skills, students can be guided to form the ability to build a data middle office and a data warehouse, and apply them to banking, education, water conservancy and other industries, forming unique big data management capabilities.

Compared with the direction of data management, the direction of data analysis has higher requirements for mathematical and statistical knowledge, and colleges and universities should set up courses such as algorithm design, mathematical logic and other mathematical knowledge improvement courses in addition to the basic courses such as higher mathematics and linear algebra, to better articulate with the courses of big data analysis and excavation such as text analysis and machine learning, and ultimately combine with the strengths of institutions to form the ability to analyse big data in financial big data analysis, big data marketing analysis and other big data analysis capabilities.

For the direction of data platform development, colleges and universities should pay attention to the improvement of computer knowledge and technology, and the courses at the knowledge improvement level should include courses such as the foundation of big data distributed platform (basic theories of Hadoop and Spark technologies) and distributed computing; at the technology improvement level, advanced development courses such as Java advanced programming language design, distributed database system (basic technologies of Mysql and Oracle technologies) and other advanced development courses should be offered.

## 6. Conclusion

The findings of this study reveal a significant misalignment between the talent cultivation objectives of Chinese universities and the explicit skill demands of the big data job market. Specifically, while over half (52%) of the analyzed programs emphasized ‘data application ability’ as a goal, a much smaller proportion (11%) of graduation requirements translated this goal into concrete, measurable skills. This gap underscores a critical challenge in the current educational paradigm. To address this disconnect, this study, leveraging a novel integration of content analysis, social network analysis (SNA), and the BERTopic-TOPSIS model, identified three distinct, data-driven employment pathways for graduates: Data Management, Data Analysis, and Data Platform Development. These pathways are not merely theoretical constructs but are derived from the quantitative mapping of industry demand onto academic supply. Consequently, our primary contribution is a set of targeted optimization proposals grounded in this supply-demand logic. We provide specific recommendations for: Enhancing general knowledge and compulsory courses to cover the common intersection of skills required across all pathways; Streamlining core course clusters for each specialized direction (e.g., adding data governance and data middle-office construction courses for the Data Management path; strengthening advanced mathematical and statistical courses for the Data Analysis path); Refining graduation requirements and training objectives to operationalize vague competencies into precise, actionable learning outcomes that mirror the competencies highlighted in high-value job postings.

In addition, this study also has limitations in terms of both the object of study and the evaluation indicators. This study mainly focuses on Chinese cases and the selection of objects is mainly limited to Chinese universities and enterprises. It does not involve foreign universities and enterprises, so the conclusions obtained are limited. This study’s analysis of curriculum commonalities is based on publicly available training programs. Consequently, institutions that provide more comprehensive and detailed documentation may be overrepresented in the sample. This potential bias towards the practices of more transparent or resourceful universities is acknowledged as a limitation of the current work. In order to make big data management and the application of talent cultivation modes international, follow-up research is required, and subsequent studies can consider: (1) Collecting recruitment data from government agencies, institutions and research institutes by means of actual research, in order to find out the actual demand for talents in the field of government affairs and scientific research. (2) The objective is to collate the big data professional training programmes of renowned foreign universities and the recruitment requirements of globally renowned enterprises in order to identify the cutting-edge knowledge and skills in the field of big data. This will facilitate the transformation and upgrading of the domestic big data profession. (3) This study uses salary as a proxy for the importance of skills. Although bias is reduced through standardisation and weight control, the effect of factors unrelated to the importance of skills still remains. Future research could introduce a more diverse range of indicators (e.g., length of corporate recruitment and skill certification requirements) in order to develop a more comprehensive assessment system.
